# Estimation of Quality of Seam Welds in AlMgSi(Cu) Extrusion by Using an Original Device for Weldability Testing

**DOI:** 10.3390/ma17225448

**Published:** 2024-11-07

**Authors:** Marek Bogusz, Dariusz Leśniak, Józef Zasadziński, Wojciech Libura, Beata Leszczyńska-Madej, Jacek Madura, Tomasz Latos, Kamila Limanówka, Bartłomiej Płonka

**Affiliations:** 1 Faculty of Non-Ferrous Metals, AGH University of Krakow, 30-059 Kraków, Poland; bogusz@agh.edu.pl (M.B.); zas@agh.edu.pl (J.Z.); libura@agh.edu.pl (W.L.); bleszcz@agh.edu.pl (B.L.-M.); madura@agh.edu.pl (J.M.); tomaszlatos@interia.pl (T.L.); 2 Łukasiewicz Research Network—Institute of Non-Ferrous Metals, 44-121 Gliwice, Poland; kamila.limanowka@imn.lukasiewicz.gov.pl (K.L.); bartlomiej.plonka@imn.lukasiewicz.gov.pl (B.P.)

**Keywords:** AlMgSi(Cu) alloys, extrusion, porthole dies, weldability testing, seam weld quality, microstructure, mechanical properties

## Abstract

Extrusion welding of AlMgSi(Cu) alloys is carried out by using porthole dies, as a result of which hollow shapes are formed with longitudinal seam welds. In the case of the inappropriate selection of the chemical composition of the aluminium alloy or improper metal welding conditions, the weld may have reduced strength in relation to that of the base material, thus weakening the strength of structures based on aluminium extrudates. The prediction of metal welding conditions, depending on the chemical composition of the alloy, the temperature and the unit welding pressures, effectively supports the design of porthole dies, thus significantly reducing the number of necessary extrusion tests and die geometry corrections needed during its implementation in industrial practice, and consequently significantly reducing production costs. In this work, an original laboratory test device simulating the behaviour of metal in a welding chamber of a porthole die was applied to examine the ability of AlMgSi(Cu) alloys to produce high-quality joints. Two different chemical compositions of AlMgSi(Cu) aluminium alloys differing in Mg, Si and Cu contents were used: alloy no. 1A (0.68% wt. Mg, 1.04% wt. Si, 0.61% wt. Cu) and alloy no. 3A (0.8% wt. Mg, 1.21% wt. Si, 1.22% wt. Cu). The weldability tests were carried out under various welding temperatures of 450, 500 and 550 °C and under various welding pressures of 150 MPa, 250 MPa and 350 MPa. The microstructural changes in the produced welds were evaluated with the use of OM and SEM/EDS with chemical analysis in micro-areas, whereas the mechanical effects were evaluated by using a static tensile test. Samples after static tensile testing were subjected to fractographic tests to determine the nature of the fractures. The highest values of relative weld strength were obtained under the highest welding temperature of 550 °C and the highest unit welding pressure of 350 MPa: 87% for alloy number 1/1A (high-strength weld), and 62% for alloy number 6/3A (medium-strength weld). Finally, the extrusion tests were performed in industrial conditions with an examination of the EBSD structure and strength of the longitudinal welds. High values of relative weld strength for extrudates from alloy no. 1/1A and alloy no. 3A, 96% and 89%, respectively, were found, which confirmed the previous weldability testing results.

## 1. Introduction

In the welding extrusion process, the heated billet is divided into inlet channels of the porthole die, and then separate streams of metal enter the welding chamber, where they join and form a hollow section. Such extrudate contains longitudinal welds, the number of which depends on the die geometry. The specific microstructure of the welds can result in the profile having poor strength. The process parameters, such as temperature and pressure in the welding chamber, extrusion speed, and particularly porthole die design, are the most important factors in effective welding. Most investigations concern 6xxx series alloys not containing copper. Valberg [1] performed a study on the metal flow and welding conditions during the extrusion of aluminium alloys and found that the role of the welding chamber height and width in weld quality in 6082 and 7008 alloys was critical. Donati and Tomesani [2,3,4] studied weld quality for the various porthole die geometry and process parameters for 6060 and 6082 alloys. They observed that the inlet channels of the porthole die should be wide to obtain high-quality welds and a high metal exit speed. They proposed a new die of the “butterfly” type, which has been implemented successfully in industry and resulted in an increase in the metal exit speed by 20%.

Many works deal with microstructure analysis of the seam weld region. Ren et al. [5] found that relatively few and small intermetallic particles were observed in the seam weld region, while denser and larger particles were present in the matrix. Lokus [6] and Bakker [7] analysed the mechanical properties and microstructure of the welds in extruded alloys 6060 and 6082. Tensile tests for samples cut at different angles on the weld showed that fracture mechanisms depend on this angle. The metal flow and the level of required compressive stresses depend on the die geometry and the temperature of the billet. The results of FEM modelling of porthole die extrusion are presented in many works [8,9,10,11,12,13]. The main studies are focused on the role of hydrostatic pressure and temperature in a welding chamber. The results indicate that the pressure value in the welding chamber is responsible for the good quality of the welds. The pressure, in turn, is determined by the geometry of both the inlet channels and the welding chamber.

The known methods of testing the weldability of metals and alloys consist of welding the two parent materials together using hot forming processes, usually upsetting or rolling. Szczegoliewatych [14] and Cook [15] carried out weldability tests on aluminium alloys, in which samples of the alloy under testing were pressed together at a given temperature, thereby increasing the number of surfaces of the materials to be joined. In order to determine the weldability of the alloy, material specimens with welds formed at different strain levels and temperatures were subjected to a static tensile test. However, the weldability tests cited above were subject to error due to the oxidation of the weld surfaces from air access. Akeret [16] investigated the weldability of easily and hardly deformable aluminium alloys using a hot rolling process with a flat ingot cut to half its length. He subjected the samples welded by the rolling process to mechanical property tests. The weldability of aluminium alloys 6060 and 6082, investigated by rolling flat ingots of different thicknesses over a wide temperature range, was evaluated by Ceretti [17] and D’Urso [18]. They assessed the quality of the joint based on structural tests of the weld area. However, also in weldability tests based on the rolling process, oxidation of the combined surfaces occurs. The weldability of aluminium alloys has also been tested using the hot extrusion process. Akeret [19] analysed the weldability of various aluminium alloys based on the hot extrusion of a flat bar through a special bridge test die. A study of the weldability of aluminium alloys based on the extrusion of an H-shape alloy was carried out by Valberg [20]. Wantuchowski, Zasadziński and Richert [21] used a special bridge die, allowing three flat bars to be extruded with a weld spot in the middle, which made it possible to test the weldability of a given alloy. Alloys 6063, 6082, 6151 and 5052 were tested. In tests based on the hot extrusion of flat bars, the problem of oxidation of the surface of the welded samples was eliminated, but the unit pressure occurring under the bridge of the bridge die could not be clearly determined. In conclusion, the known methods of assessing the weldability of aluminium alloys do not fully reproduce the welding conditions prevailing during extrusion through porthole dies, and hence the results obtained are difficult to use in practice. There are no weldability tests in which the welding temperature, unit pressure, welding time and the amount of deformation in the weld zone can be adjusted simultaneously. A serious complication is the need to provide metal joining conditions in the test without an excess of air oxidising the surfaces of the welded metal planes. In order to effectively design tools and process parameters for the extrusion of aluminium alloys through porthole dies, a suitable test was proposed to produce an airless joint and to determine the influence of the basic process parameters determining the quality of the joint. The data obtained from the weldability test, i.e., minimum unit pressure and minimum weld temperature, can be used as a basis for designing porthole dies using numerical FEA methods.

In the work [22], a novel method was proposed to quantitatively evaluate welding quality during the three-dimensional extrusion process by combining the Forge-3D and Pressure–Time-Flow criterion (K criterion). Similar investigations were performed by Sariyarlioglu et al. [23]. Based on the proposed method, the effects of extrusion speed and welding chamber on welding quality are investigated.

A new improved dimensionless welding quality criterion that takes into account the imbalances of metal flow is proposed by the Kniazkin in [24]. The proposed criterion has been approved by industrial experiments for different types of profiles of the extruded form of the 6082 alloy. A comparative study [25] of seam weld quality was conducted using two different die designs. The modified original design of multi-container extrusion can obtain better welding quality, as evaluated by different welding criteria.

Microstructural observation, mechanical property testing and welding quality analysis were performed continuously on different positions of a longitudinal weld [26]. The metal flow law in the unsteady stage during the porthole die extrusion process was studied, and the interfacial bonding mechanism of the longitudinal welds in the unsteady zones of the profiles was proposed. The effect of ram speed on extrusion welding was investigated experimentally and analysed numerically [27]. Increasing ram speed negatively influences the seam weld quality of AA6063. With increasing ram speed, in numerical simulations, normal pressure P and effective stress σ increased.

As seen from the above review, research is aimed at the investigation of the weld quality of a finished profile fabricated in industrial conditions. There are few papers dealing with laboratory investigations focused on predicting the most favourable conditions for the best weld quality. In [28], thin-walled, wide AA6063 hollow profiles were extruded by three-container extrusion technology under different temperature and speed conditions. The quality of the welds that formed between the adjacent billets was experimentally evaluated by tensile tests of specimens at different positions of the extrudates. Wedge and bulge expansion tests were compared in determining the seam weld strength in a tubular profile extruded at two ram speeds [29]. In the wedge test, the expansion was determined by moving a conical punch into the tube until the specimen fractured.

The investigators tried to analyse the welding conditions by specific laboratory tests. Unfortunately, these studies were useless because they avoided important factors and phenomena occurring in the real extrusion process. Particularly important is that in real processes, welding occurs without contact with air.

Worth noting is the fact that there is a lack of papers dealing with predicting the weldability of AlMgSi alloys containing Cu. This element is known to inhibit the joining process during extrusion, as with AlCuMg alloys. Therefore, researching the weldability of these alloys seems to be necessary.

In this work, an innovative way of predicting the seem weld quality of profiles from AlMgSi(Cu) alloys including an alloy with an increased Cu content of above 1.2% is presented. The original method is based on the implementation of a patented laboratory device [30] for analysing the welding process, which allows for the exact modelling of conditions existing in a porthole die. The industrial verification confirmed the laboratory predictions.

## 2. Materials and Methods

### 2.1. Characterisation of AlMgSi(Cu) Alloys

The samples for the weldability tests were made from AlMgSi(Cu) alloy ingots with 2 different chemical compositions, alloy 1/1A and alloy 6/3A, made using a semi-continuous vertical casting (DC) method. The chemical compositions of the alloys are shown in Table 1. Properly performed 3-stage homogenisation on the alloys in question (475 °C/2 h + 530 °C/2 h + 545 °C/2 h) produced a homogeneous material structure with fine particles of strengthening phases, resulting in a significant increase in solidus temperature (Table 2).

### 2.2. Device for Predicting the Seam Weld Quality of Extrudates

**Figure 1:**
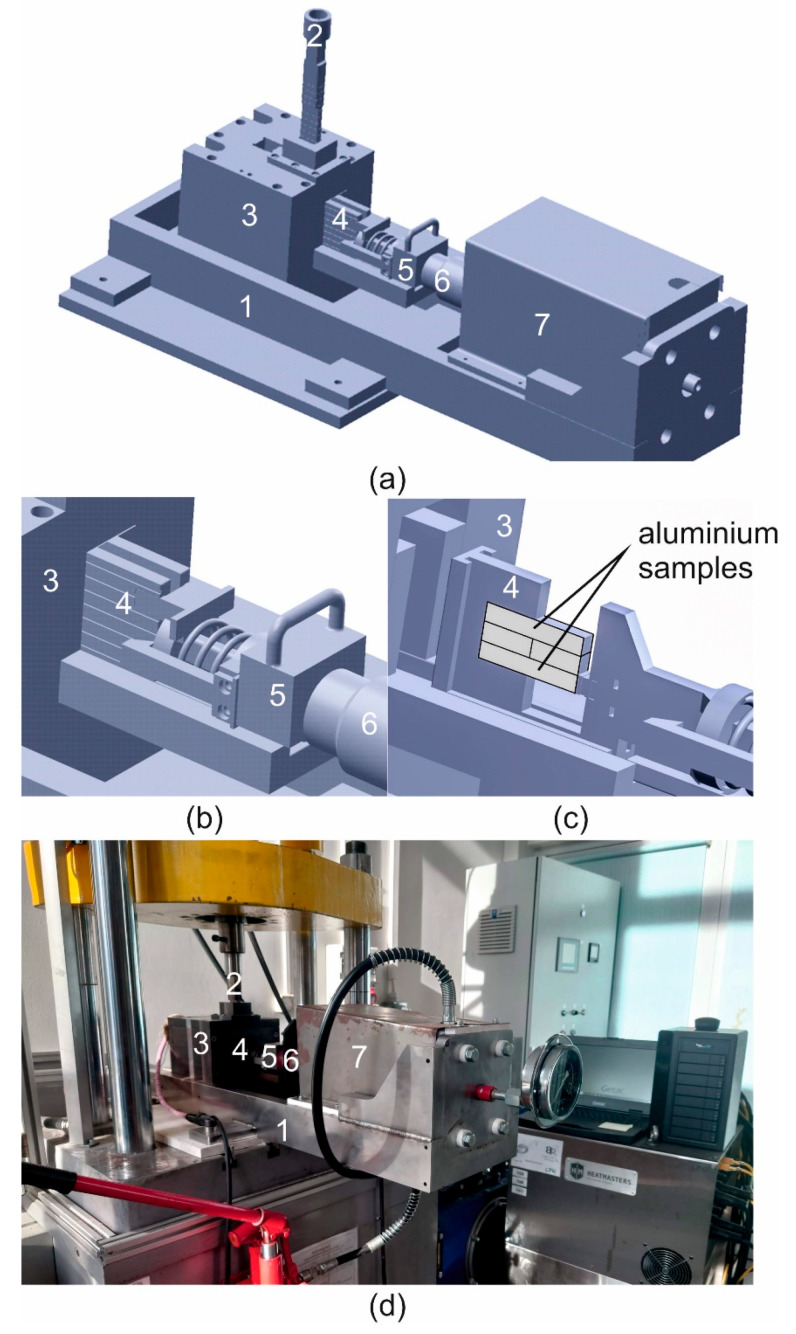
Device for predicting the seam weld quality of extrudates: 3D model (**a**–**c**) and laboratory device (**d**). The numbers represent the following: 1—main construction ram; 2—upper shearing punch; 3—heating cell; 4—welding cartridge tool; 5—adapter; 6 –hydraulically driven stem; 7—hydraulic cylinder (Patent No PL243256B1, AGH Krakow, 2022 [30]).

A new patented device for predicting the seam weld quality of extrudates was designed and produced (Figure 1 [22]). This device enables the modelling of welding conditions without air for the design of porthole dies of a hollow shape. The weldability test consists of cutting two aluminium samples and pressing them under different temperature and pressure conditions. First, the aluminium samples placed in the welding cartridge tool (4) are heated in the heating chamber (3) to the desired welding temperature. Then, shearing of samples is performed by the upper stem (2), and axial pressure is exerted by using a hydraulic pressing stem (5).

**Figure 2:**
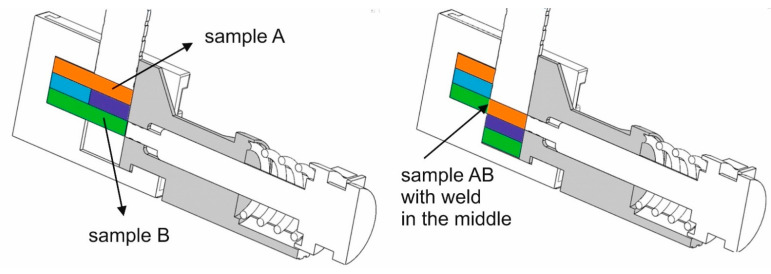
Setup of samples in the toolbox in the starting position (**left**) view and in the ending position (**right**): orange and green colours mean samples from aluminium alloy; blue and purple colours mean counter-samples from steel.

**Figure 3:**
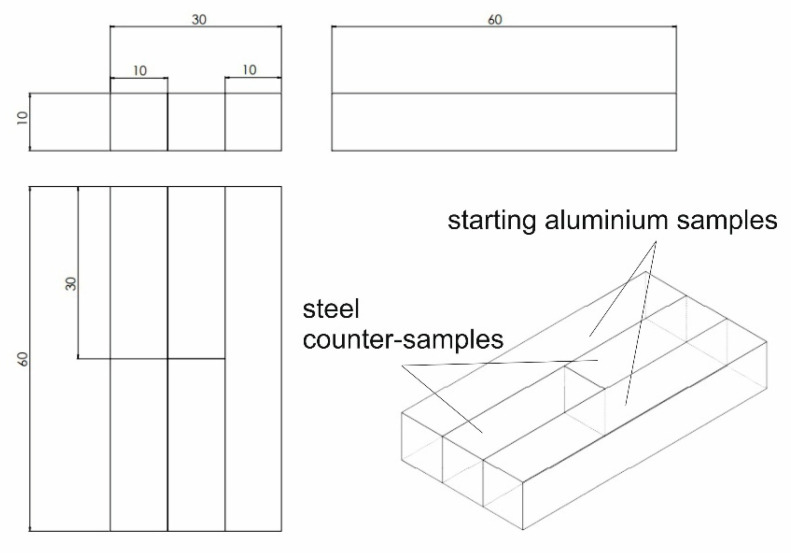
Geometry of samples for weldability tests in the starting position.

**Figure 4:**
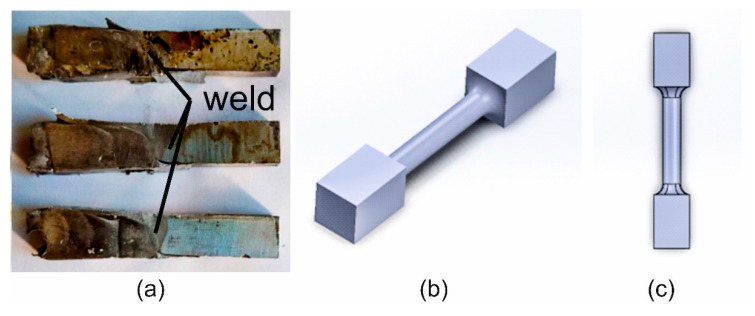
Aluminium samples with the weld in the middle after weldability testing: example samples removed from the welding cartridge tool after the weldability test (**a**) and 3D CAD models indicating the geometry of samples prepared for the tensile test (**b**,**c**).

The initial sample dimensions were of 60 × 10 × 5 mm (Figure 2 left). The key dimensions are shown in Figure 3. We placed one aluminium specimen, specimen ‘A’, on top in the cassette and the other, ‘B’, on the bottom. Between sample ‘A’ and ‘B’, we placed the counter samples (left and right). During the test, the toolbox (4) was pushed into the heating cell (3), the top punch (2) was driven to make contact, and the specimens were compressed with a force of 500 kG by the side punch equipped with a spring pressure system (5). After heating the specimens to the specified temperature (with the time depending on the set temperature), the top punch (2) cut specimens ‘A’ and ‘B’ by displacing the right side with the right counter punch by a distance of 20 mm, thus obtaining one specimen, ‘AB’, and two halves of specimens ‘A’ and ‘B’ (Figure 2 right). The newly created specimen, ‘AB’, which had the left side of specimen ‘B’ and the right side of specimen ‘A’ in its structure, was compressed with a side punch (5). After the test, the new two-piece specimen, ‘AB’, was prepared into a 6 mm diameter base sample with a length of 60 mm and subjected to a tensile test (Figure 4).

The variables in the weldability study of the AlMgSi(Cu) alloy included the chemical composition of the alloy: Mg, Si and Cu—alloy No 1/1A (0.68 wt.% Mg, 1.04 wt.% Si, 0.61 wt.% Cu), alloy No 6/3A (0.8 wt.% Mg, 1.21 wt.% Si, 1.22 wt.% Cu), and alloy No. 7/3B (0.8 wt.% Mg, 1.22 wt.% Si, 1.41 wt.% Cu). Different welding temperatures (450, 500 and 550 °C) and different welding unit pressures (150, 250 and 350 MPa) were also applied. Th number of measurements taken during the weldability test was 3 for the given aluminium alloy and for the given welding temperature and welding pressure. Nine variants were tested for a given aluminium alloy at 3 different welding temperatures and 3 different welding pressures. Finally, we had 27 measurements for the weldability test on the given alloy.

### 2.3. Extrusion Process

**Figure 5:**
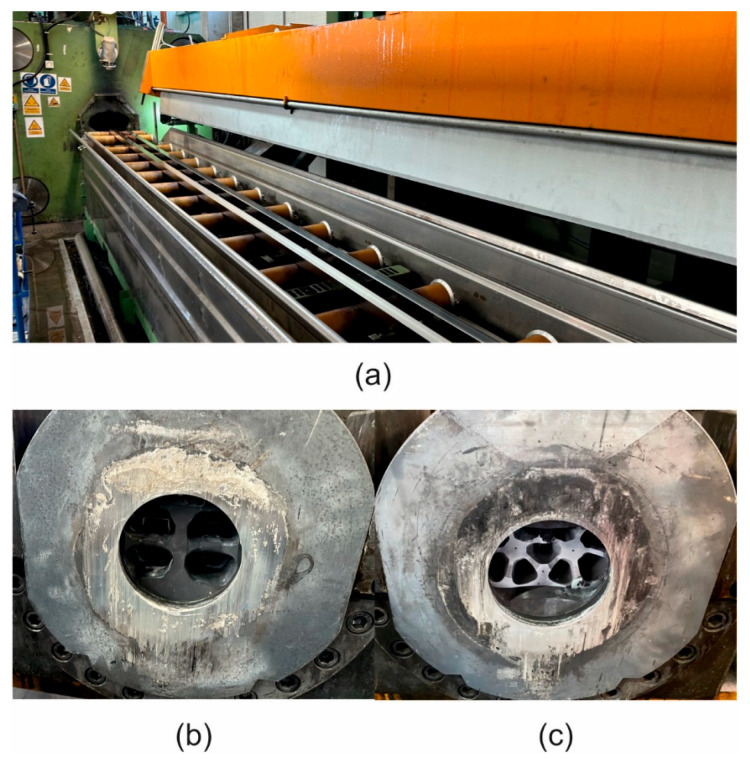
The run-out table of the 25 MN 7-inch extrusion press (**a**) and porthole dies of different constructions: the porthole die for the production of the tube measuring Ø 60 × 3 mm from alloy 1A (**b**), and the porthole die for the production of the profile measuring 40 × 40 × 3 mm from alloy 3A (**c**).

**Figure 6:**
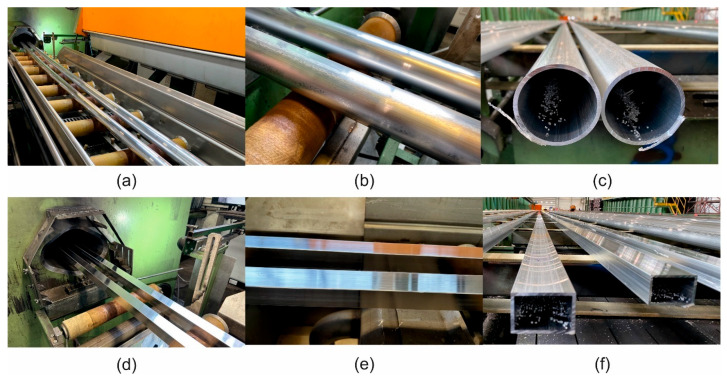
The extrudates produced from the AlMgSi(Cu) alloys with different Cu contents in industrial conditions: tubes measuring Ø 60 × 3 mm while extruding alloy 1A; (**a**) tubes measuring Ø 60 × 3 mm while extruding alloy 1A—surface appearance (**b**); cross-section of tubes measuring Ø 60 × 3 mm while extruding alloy 1A (**c**); profiles measuring 40 × 40 × 3 mm while extruding alloy 3A (**d**); profiles measuring 40 × 40 × 3 mm while extruding alloy 3A—surface appearance (**e**); cross-section of profiles measuring 40 × 40 × 3 mm while extruding alloy 3A (**f**).

Extrusion tests of the tube measuring Ø 60 × 3 mm from alloy 1A and the profile measuring 40 × 40 × 3 mm from alloy 3A were performed by using the 2-hole porthole dies on the 7-inch press with a 25 MN load (Figure 5). The conditions of the extrusion tests were as follows: billet temperature—480 °C; container temperature—450 °C; extrusion ratio—45.7 and 55.2, respectively,; and metal exit speed—8 m/min and 6 m/min, respectively. Extrudates were subjected to intensive cooling on the run-out table by using water and then to artificial ageing at 175 °C/8 h. The surface quality of extrudates was inspected to prevent cracking or tarnishing (Figure 6). The metal exit speed for each alloy was determined. From the extruded and aged profiles obtained, the samples were prepared for tensile testing.

### 2.4. Methodology of the Microstructural and Mechanical Examination

**Figure 7:**
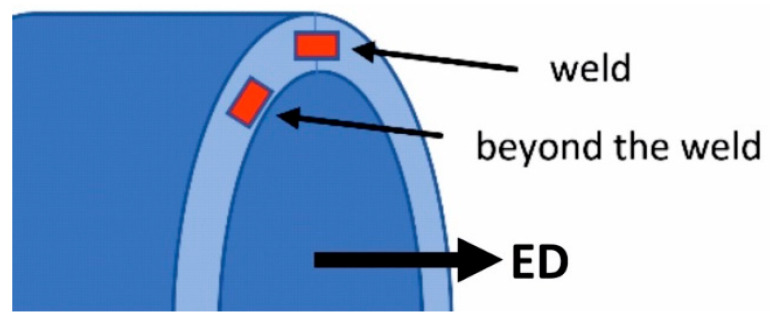
Sampling locations for structure and mechanical property tests.

The sampling method for further examination of the microstructure and mechanical properties of extruded profiles is illustrated in Figure 7. Samples for microscopic analysis were embedded in resin, ground mechanically with graded sandpaper, and subsequently polished in two stages. Initial polishing was performed using a diamond paste suspension, followed by final polishing with a colloidal silicon oxide suspension. For microstructure observation using a light microscope, samples were anodised in Barker’s reagent, consisting of 100 mL H_2_O and 2 mL HBF_4_. The microstructure was examined using light microscopy (OLYMPUS GX51 microscope, Tokyo, Japan) and scanning electron microscopy (Hitachi SU 70 microscope, Tokyo, Japan). Additionally, micro-area chemical composition analysis was conducted using energy-dispersive X-ray spectroscopy (EDS) (Thermo Fisher Scientific, Waltham, MA, USA) to identify the distribution of alloying elements within the grains.

Grain diameter measurements were taken both within the weld area and outside it, applying the average chord method. Typically, specimens with the weld located centrally were prepared for static tensile testing to evaluate joint quality by comparing the mechanical properties of welded and non-welded (reference) samples. Key mechanical properties—yield strength (YS), ultimate tensile strength (UTS), and elongation (A%)—were assessed using the INSPECT 100 testing machine, which has a maximum load capacity of 100 kN. For accuracy, each sample group underwent a minimum of three tests, with the final results being the mean values of these trials.

The crystallographic orientation was analysed using the EBSD technique, equipped with the Velosity Plus camera and the Inspect F50 scanning electron microscope (originally produced by FEI Company, now part of Thermo Fisher Scientific, Waltham, MA, USA). Data from the EBSD analysis were processed with Apex 3.0 software and EDAX TSL OIM Analysis 8.0 (EDAX, Inc., Mahwah, NJ, USA), referencing the ICCD 2011 PDF database. Samples were polished by standard metallographic techniques and subsequently underwent ion milling with the Leica EM RES101 device (Leica Microsystems, Wetzlar, Germany). The tests were conducted at an accelerating voltage of 20 kV, with a working distance (WD) of 15 mm. The measurement step was set to 0.2 µm, while the mapping area covered 320 × 250 µm. The disorientation angle ranges were categorised and mapped: red and green colours in the disorientation maps indicate low-angle grain boundaries or subgrain boundaries with disorientation angles of several degrees. Specifically, red marks boundaries with disorientation angles between 1° and 5°, green marks those between 5° and 15°, and blue marks high-angle grain boundaries with disorientation angles above 15°.

## 3. Results

### 3.1. Weldability Testing

**Figure 8:**
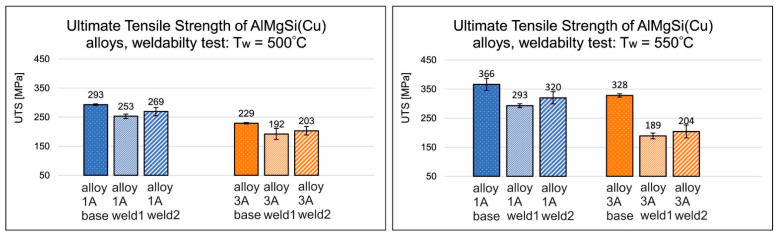
Influence of welding temperature on the ultimate tensile strength, UTS, of samples after the weldability testing of AlMgSi(Cu) alloys 1A and 3A: welding temperature of 500 °C (**left**) and welding temperature of 550 °C (**right**).

Figure 8 shows the obtained ultimate tensile strength (UTS) values for welded and reference (base) samples made of selected AlMgSi(Cu) alloys: alloy 1/1A and alloy 6/3A under welding temperatures of 500 °C and 550 °C and welding pressures of 250 and 350 MPa. As indicated by the data in the graphs, the highest strength properties were obtained for the non-welded/base specimens of AlMgSi(Cu) alloy 1/1A and 6/3A under a welding temperature of 550 °C and a welding pressure of 350 MPa: 366 and 328 MPa, respectively. In general, also for welded specimens, the highest UTS values were obtained for the highest of the analysed welding temperatures (550 °C) and the highest of the analysed welding pressures (350 MPa)—320 MPa and 204 MPa, respectively. In the case of alloy 1/1A, high-quality, high-strength welds were be obtained, i.e., welds with a relative strength of min. 87%, while alloy 6/3A yielded medium-strength welds (relative strength in the range of 62%). However, for alloy 6/3A, high-strength welds were produced at a welding temperature of 500 °C, with a relative strength of 89% under a welding pressure of 350 MPa.

**Figure 9:**
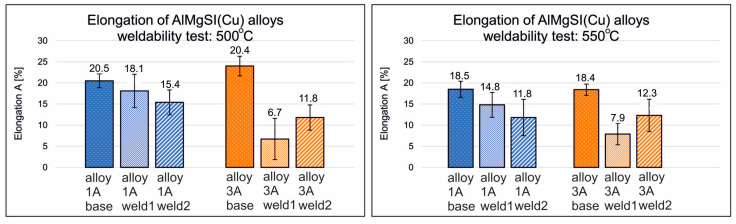
Influence of welding temperature on the elongation A of samples after the weldability testing of AlMgSi(Cu) alloys 1A and 3A: welding temperature of 500 °C (**left**) and welding temperature of 550 °C (**right**).

**Figure 10:**
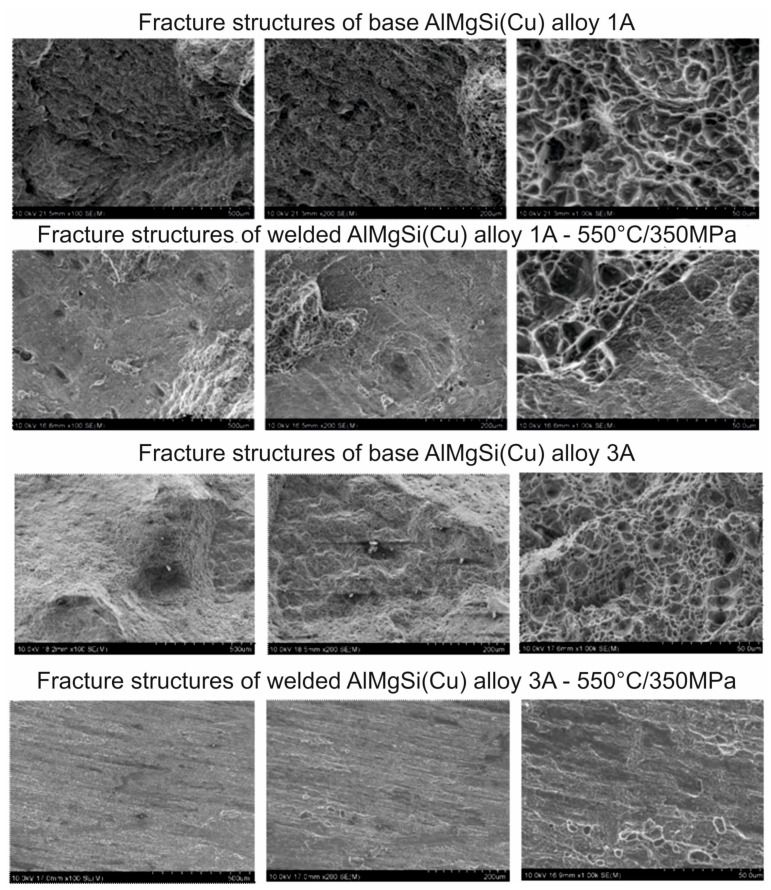
Fractured samples after uniaxial tensile test for AlMgSi(Cu) alloys nr 1A and 3A for base material zone and weld zone; T = 550 °C, p = 350 MPa.

Regarding the ductility of the welded samples, very-high-percentage elongation was obtained for AlMgSi(Cu) alloy 1/1A under welding temperatures of 500 and 550 °C, which indicates an already well-plasticised joint (Figure 9). For a welding temperature of 550 °C, elongation values of 12–15% were obtained for alloy 1/1A and 8.5–12% for alloy 6/3A. This is confirmed by the results of fractographic studies of the welded specimens in relation to the base specimens, where for the aforementioned welding conditions for individual alloys from 1/1A to 6/3A, fractures with a ductile character or that were predominantly ductile as well as brittle were observed (Figure 10). The fractures of the base (aged) specimens, regardless of the heat treatment conditions used, were transcrystalline plastic in nature, characterised by the occurrence of numerous dimples and hills. In the case of alloy 1/1A, a plastic fracture was observed, and for alloy 6/3A, after the welding process, for some process conditions, the fractures were characterised by low surface development, which may be indicative of poor bonding during the welding process.

**Figure 11:**
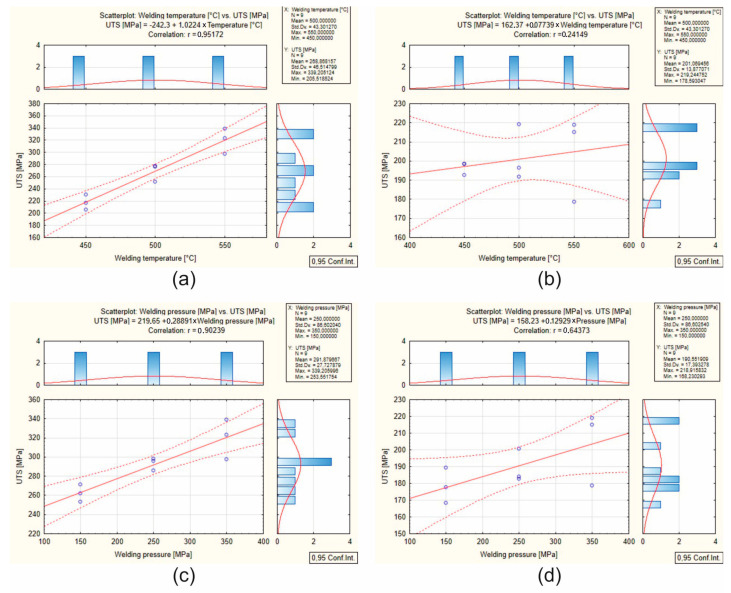
Scatter plots for the weldability test: UTS for AlMgSi(Cu) alloys no 1A (**a**,**c**) and 3A (**b**,**d**)—(**a**) correlation of UTS–welding temperature (450 °C, 500 °C and 550 °C) for alloy 1A; (**b**) correlation of UTS–welding temperature (450 °C, 500 °C and 550 °C) for alloy 3A; (**c**) correlation of UTS–welding pressure (150 MPa, 250 MPa and 350 MPa) for alloy 1A; (**d**) corelation of UTS–welding pressure (150 MPa, 250 MPa and 350 MPa) for alloy 3A (dotted lines are border lines and solid lines are medium lines).

Figure 11 shows scatter plots of the results for AlMgSi(Cu) alloy samples 1A and 3A: Figure 11a, correlation of UTS and welding temperature for alloy 1A; Figure 11b, correlation of UTS and welding temperature for alloy 3A; Figure 11c, correlation of UTS and welding unit pressure for alloy 1A; Figure 11d, correlation of UTS and welding unit pressure for alloy 3A. Based on the plotted graphs, the correlation coefficients were analysed. Specifically, correlation coefficients from 0.6 to 1.0 were considered. A strong correlation can be found for the following variables for alloy 1A: UTS–welding temperature (correlation coefficient 0.95); UTS–welding unit pressure (correlation coefficient 0.90). A moderate positive correlation for alloy 3A was obtained for the statement UTS with unit weld pressure (correlation coefficient 0.64). In contrast, a low positive correlation for alloy 3A was obtained for the statement UTS with unit weld pressure (correlation coefficient 0.24). In practice, this implies a large increase in weld strength due to the increasing unit weld pressure, for both alloy 1A and alloy 3A. In the case of alloy 3A, an increase in the unit welding pressure only had a moderate effect on the increase in weld strength, and an increase in welding temperature had only a small effect on the increase in weld strength. The above statistical analysis of the correlation of the weldability test results also indicates full control in achieving adequate weld quality for alloy 1A by adjusting the temperature and welding stress parameters. For alloy 3A containing more Cu in the chemical composition, this control is at a much lower level.

**Figure 12:**
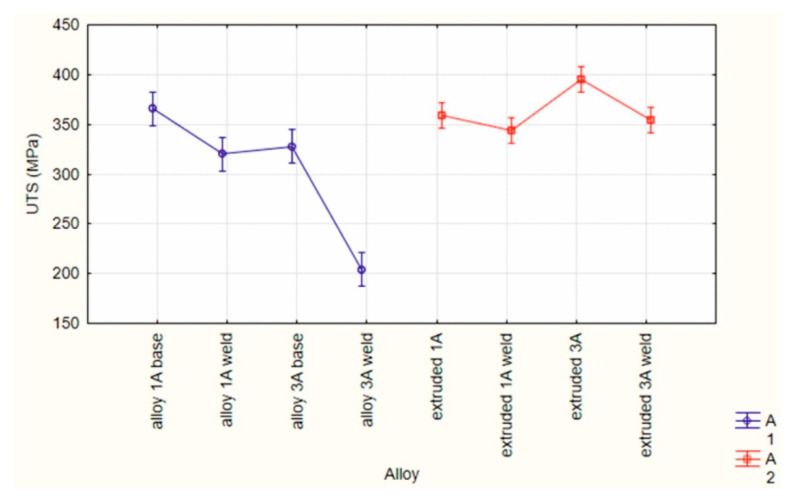
Results of ANOVA for the weldability test and extrusion process: UTS for welded and unwelded samples from AlMgSi(Cu) alloys no 1A and 3A.

An ANOVA statistical analysis was also carried out for welded and unwelded samples of AlMgSi(Cu) alloys numbered 1A and 3A in the weldability test and the industrial extrusion test (Figure 12). In the graph shown, it can be seen that slightly higher UTS values were obtained in the extrusion test, and at the same time, similar values were obtained for the samples in the welding area and outside the weld. In the weldability tests, a significantly lower UTS was observed for the welded sample compared to the non-welded sample for alloy 3A. This may indicate insufficient precipitation of the alloy during its release from the tool cassette during the weldability tests, which later translated into much lower UTS values of the samples after final artificial ageing.

**Figure 13:**
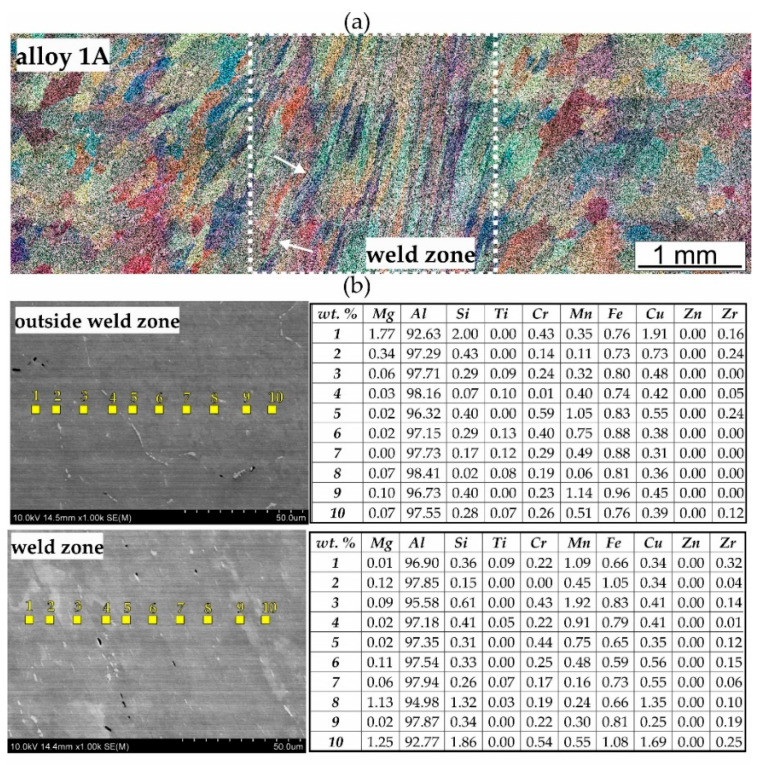
Microstructure of the laboratory-welded 1A alloy, with welding at T = 550 °C and p = 350 MPa: (**a**) cross-section through the sample, LM; (**b**) results of the chemical composition analysis on the grain cross-section, SEM/EDS.

**Figure 14:**
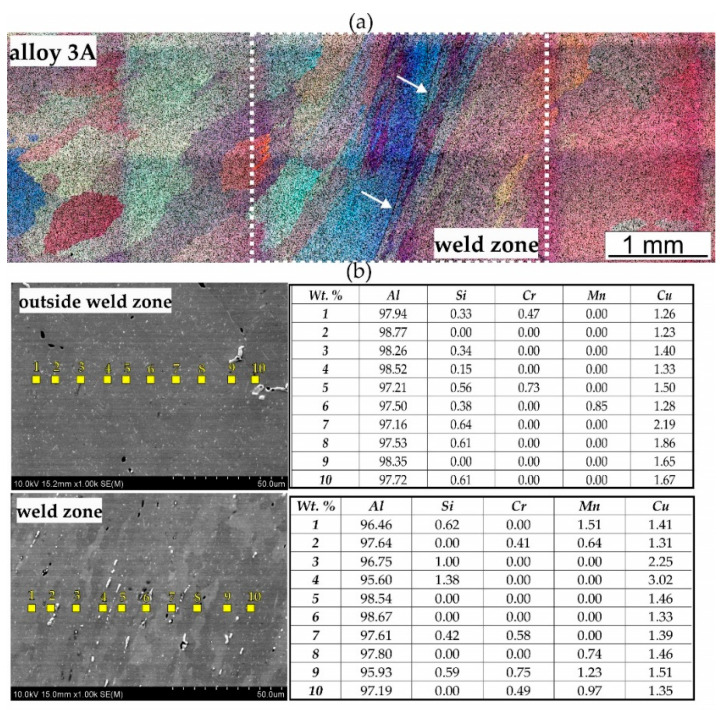
Microstructure of the laboratory-welded 3A alloy, with welding at T = 550 °C and p = 350 MPa: (**a**) cross-section through the sample, LM; (**b**) results of the chemical composition analysis on the grain cross-section, SEM/EDS.

The tests on the ability to weld were conducted for various welding temperatures, 450, 500, and 550 °C, and for various welding pressures of 150 MPa, 250 MPa, and 350 MPa. The microstructural changes in the produced welds were evaluated with the use of optical microscopy (OM) and scanning electron microscopy (SEM/EDS) with chemical analysis in micro-areas. Figure 13 and Figure 14 present representative images showing the microstructure both within and outside the weld. The best results were obtained from specimens welded under laboratory conditions at 550 °C and 350 MPa, and because of that, the article includes the microstructural results for these specimens.

Figure 13a presents the diverse microstructure of alloy 1A, clearly delineating the weld zone from the surrounding areas. The weld zone is characterised by elongated grains and an irregular microstructural orientation, while the grains outside the weld exhibit a regular, near-axial shape. Figure 13b shows the microstructure examined by scanning electron microscopy (SEM) along with point chemical composition analysis using energy-dispersive spectroscopy (EDS) expressed in weight percent. The accompanying table lists the elemental contents, including Mg, Al, Si, Ti, Cr, Mn, Fe, Cu, Zn, and Zr. Outside the weld zone, the microstructure is more homogeneous. The magnesium (Mg) content ranges from 0.02% to 1.77%, and the aluminium (Al) content is notably high, with values between 92.63% and 97.73%. Silicon (Si) is present in amounts ranging from 0.07% to 2.00%, indicating the presence of silicon-containing phases. Small amounts of other elements, such as Fe, Mn, and Zn, are also detected, though their concentrations are relatively low. In the weld zone, noticeable changes in microstructure are observed. The Mg content ranges from 0.07% to 1.25%, reflecting differences in chemical composition compared to the area outside the weld zone. The Al content remains high, ranging from 96.90% to 97.71%, while that of Si varies from 0.15% to 1.86%. Additionally, higher concentrations of Mn and Zn are found in the welding area compared to the surrounding area, which may result from localised changes during the welding process.

The microstructure of alloy 3A exhibits significant variation between the areas outside the weld zone and the weld zone itself. The microstructure outside the weld zone features a coarser grain structure that appears more equiaxed and randomly oriented. In contrast, the weld zone reveals a more elongated and refined grain structure (Figure 12). Figure 11b displays the microstructure analysed by SEM along with point chemical composition analysis using energy-dispersive spectroscopy (EDS), expressed in weight percent. The accompanying table lists the elemental contents: Mg, Al, Si, Ti, Cr, Mn, Fe, Cu, Zn, and Zr. Outside the weld zone, the microstructure is more homogeneous. The aluminium (Al) content remains high and consistent across the measured points, ranging from 97.16% to 98.77%. The amount of silicon (Si) is generally low but noticeable, with values reaching up to 0.64%. Copper (Cu) levels are relatively higher in some areas, such as 1.40% and 2.19%. Manganese (Mn) is also present in notable amounts, with values ranging from 0.85% to 1.86%. The chromium (Cr) and silicon (Si) contents are relatively low, though Cr shows a slight increase toward the weld zone boundary. In the weld zone, the aluminium (Al) content is slightly reduced compared to that in the outside zone, varying from 95.60% to 97.61%. Silicon (Si) displays a significant increase at certain points, reaching up to 1.38%, likely indicating a localised increase due to welding effects. The copper (Cu) content remains high, reaching up to 1.51% in some regions, suggesting a concentration shift within the weld zone. Manganese (Mn) values also increase within the weld zone, reaching up to 1.51%, and indicating that the welding process may have caused a redistribution of Mn across the weld interface.

A characteristic feature of the microstructure of both welds is the presence of numerous precipitates within the grains. The number of precipitates is higher in alloy 3A due to its greater content of alloying elements. Measurements of the weld zone width indicate that it is similar for both alloys, averaging about 2.4 mm. The comparison between alloy 1A and alloy 3A highlights differences in the grain structure and elemental distribution, with alloy 3A exhibiting a higher segregation of copper and a finer microstructure in the weld zone. These factors may influence the mechanical performance of alloy 3A, in contrast with that of the alloy 1A, particularly regarding weld strength and corrosion resistance. Furthermore, there are no visible discontinuities in the microstructure of weld (1A or 3A), indicating that the weld interfaces are free from defects such as voids or cracks. The grain size of samples welded under laboratory conditions was determined using the mean chord method. Measurements were taken inside and outside the welding zone. In each case, a minimum population of 200 grains was tested. The average grain diameter after weldability tests of alloy 1A in the weld zone was 155 μm. The size of the measured grains ranged from 55 to 264 μm. Outside the weld zone, the grains were larger, with an average diameter of 218 μm and measured grain sizes ranging from 60 to 322 μm. For alloy 3A, the grains were significantly larger, and the grains were also more variable in size than for alloy 1A. In the weld area, the average grain diameter was 384 μm. The size of the measured grains ranged from 130 to 960 μm. Outside the welding area, the grain size was larger, with an average diameter of 627 μm and a measured grain size in the range 146–1220 μm.

### 3.2. Extrusion Trials

**Figure 15:**
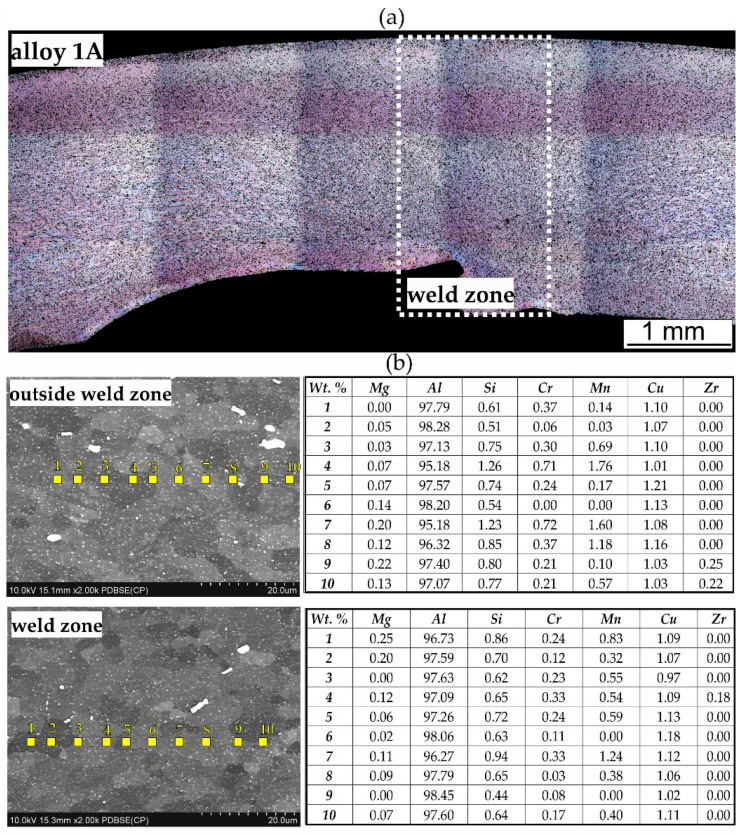
Microstructure of the extruded profile of alloy 1A: (**a**) cross-section through the profile, LM; (**b**) results of the chemical composition analysis on the grain cross-section, SEM/EDS. Data in table indicates that outside the weld zone, the magnesium (Mg) content ranges from 0.22% to 0.46%. The aluminium (Al) content varies from 95.54% to 97.16%. Silicon (Si) is present in amounts ranging from 0.54% to 1.15%, while copper (Cu) ranges from 1.53% to 1.97%. Chromium and other elements, such as manganese (Mn) and zirconium (Zr), are present in small amounts. In the weld zone, the concentrations of these elements remain at similar levels (Figure 16b). The local increase in the concentration of a given element is likely related to the fact that the compositional analysis was performed at the site of the precipitation.

**Figure 16:**
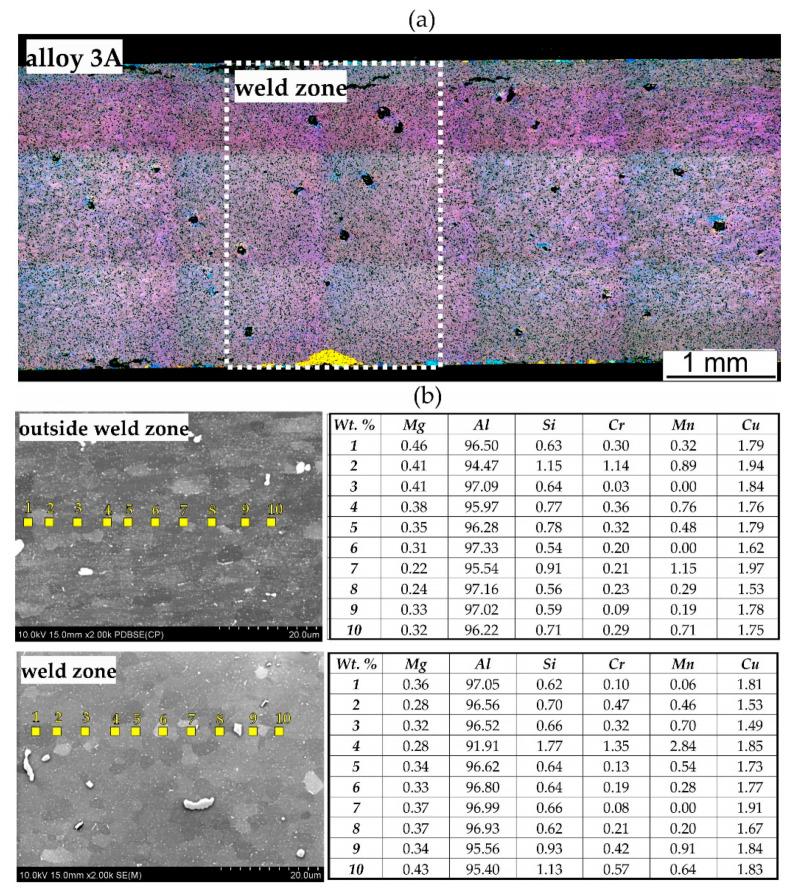
Microstructure of extruded profile of alloy 3A: (**a**) cross-section through the profile, LM; (**b**) results of the chemical composition analysis on the grain cross-section, SEM/EDS.

**Figure 17:**
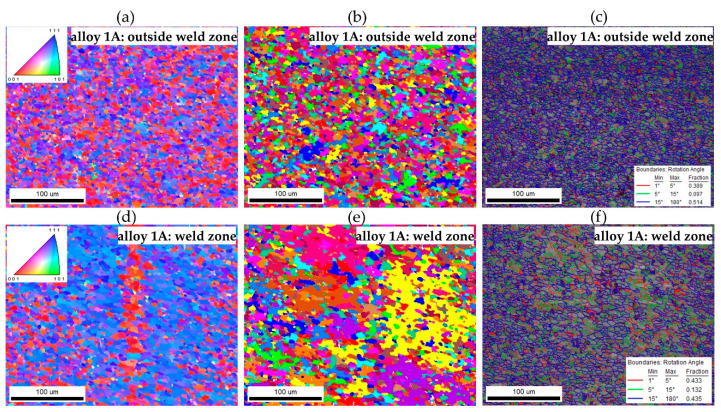
EBSD analysis of the extruded profile: alloy 1A (**a**–**c**) outside weld the zone area, and (**d**–**f**) in the weld zone; (**a**,**d**) EBSD maps showing changes in orientation (IPF); (**b**,**e**) EBSD maps showing grains in the microstructure (grain contrast); (**c**,**f**) maps showing the types of grain boundaries (the boundaries with a large angle are marked in blue, and the boundaries with a small angle are marked in red and green).

Figure 15, Figure 16 and Figure 17 show microstructure of the extruded profiles. The tests conducted did not reveal any significant differentiation in the microstructure of the profiles between the welding area and the areas outside the welding zone. The grain size in these profiles is significantly finer compared to samples welded under laboratory conditions. The results of the chemical composition analysis of the profile extruded from alloy 1A indicate that outside the weld zone, the magnesium (Mg) content is low, ranging from 0% to 0.22%. The aluminium (Al) content ranges from 95.18% to 97.79%. Silicon (Si) is present in amounts ranging from 0.51% to 1.26%, while copper (Cu) ranges from 1.03% to 1.21%. Chromium and other elements, such as manganese (Mn), and zirconium (Zr), are present in small amounts. In the weld zone, slightly higher magnesium values are observed, reaching up to 0.25%. The aluminium content remains relatively consistent, ranging from 96.73% to 98.60%. The silicon values in the weld zone are lower than those outside, varying from 0.64% to 0.94%. The copper content in the weld zone is comparable to that outside, ranging from 0.94% to 1.18% (Figure 15b).

**Figure 18:**
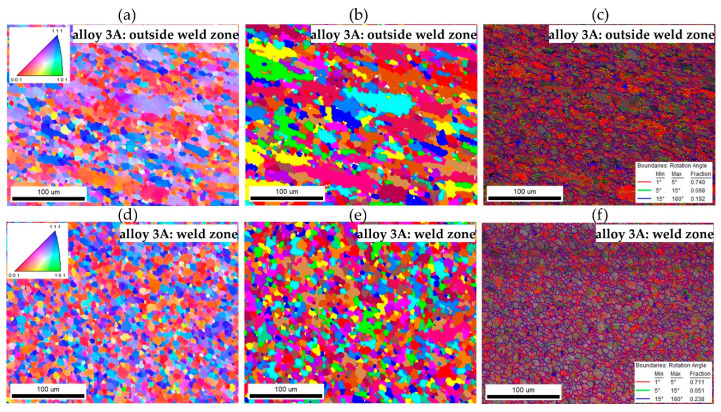
EBSD analysis of the extruded profile; alloy 3A (**a**–**c**) outside the weld zone area and (**d**–**f**) in the weld zone; (**a**,**d**) EBSD maps showing changes in orientation (IPF); (**b**,**e**) EBSD maps showing grains in the microstructure (grain contrast); (**c**,**f**) maps showing the types of grain boundaries (the boundaries with a large angle are marked in blue, and the boundaries with a small angle are marked in red and green).

**Figure 19:**
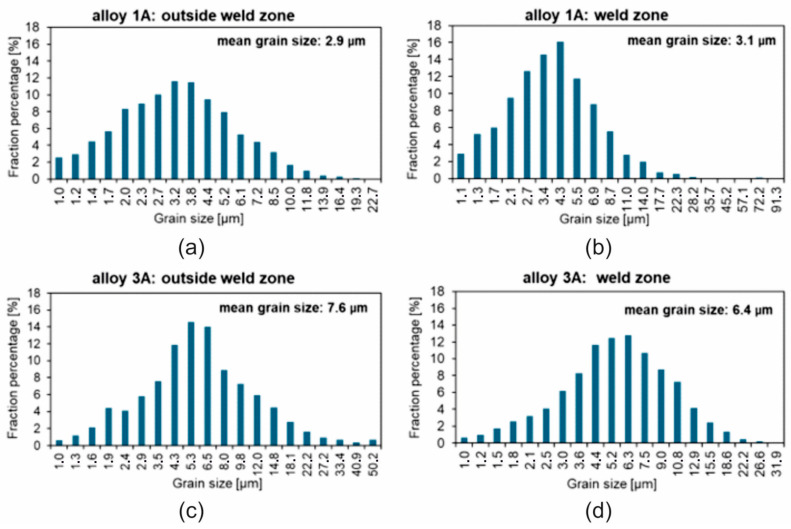
Results of statistical analysis of grain size: (**a**,**b**) alloy 1 A ((**a**)—outside the weld zone; (**b**)—weld zone); (**c**,**d**) alloy 3A ((**c**)—outside the weld zone; (**d**)—weld zone).

The EBSD tests confirmed a homogeneous, fine-grained microstructure in the cross-section of the tested profiles. No defects, such as cracks or discontinuities, were observed, nor were any coarse crystalline structures detected. The grains, examined using the SEM-EBSD method in the cross-section transverse to the extrusion direction, were predominantly equiaxed in shape, both within and outside the weld zone. However, for the extrusion profile from alloy 3A, larger grains were observed locally in the microstructure (Figure 17 and Figure 18). The average grain sizes both within and outside the weld zone, determined using the EBSD method for the profile extruded from alloy 1A, are comparable, at approximately 3 µm (Figure 17). In contrast, the profile extruded from alloy 3A is characterised by a larger grain size and variation in size between the weld zone and the surrounding area. In the weld zone, the average grain size is 6.4 µm, while outside the weld zone, it increases to an average of 7.6 µm (Figure 18). These differences are attributed to the presence of individual large grains in this area, as also confirmed by the grain size distribution histograms (Figure 19a–d).

For profiles extruded under industrial conditions, the grain size was determined from the results of the EBSD analysis. Grains with a high misorientation angle, i.e., above 15°, were taken for analysis. In the case of the alloy 1A extrusion profile, approximately 4600 grains were taken outside the weld area for analysis, with approximately 2100 grains in the weld area, while in the case of the alloy 3A extrusion profile, the average grain diameter outside the weld area was determined from approximately 1300 grains, and that in the weld area was determined from approximately 1800 grains. The average grain diameter in profiles extruded under industrial conditions was significantly smaller than in samples welded under laboratory conditions. In the case of profiles extruded from alloy 1A, the average grain diameters in and outside the welding area was comparable at around 3 μm. The spread of results was also smaller than for the samples after the weldability test. In the area outside the weld zone, the size of the measured grains ranged from 1 to 23 μm. In the weld zone, 99% of the population of measured grains was in the range 1–23 μm, with only single grains of 25–91 μm observed in the microstructure (Figure 19). The average grain diameters in the profiles extruded from alloy 3A were 6.4 and 7.6 μm in and outside the weld zone, respectively. In the area outside the welding zone, the size of the measured grains ranged from 1 to 50 μm; with 97% of the population of measured grains falling within the range in the area outside the welding zone, the size of the measured grains ranged from 1 to 22 μm. In the weld zone, the size of the measured grains ranged between 1 and 32 μm, with 99% of the population of measured grains falling within the range of 1–18 μm (Figure 19).

**Figure 20:**
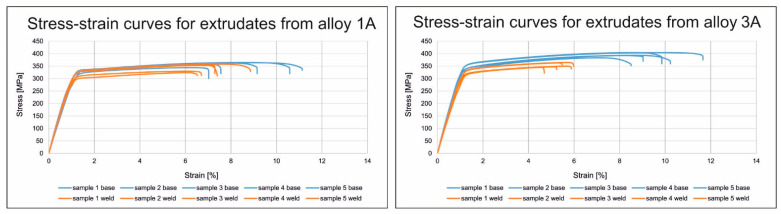
Stress–strain curves for AlMgSi(Cu) alloy extrudates from static tensile test: the base material and the material from the weld zone.

Stress–strain curves from static tensile test for the base material and the material from the weld zone are presented in Figure 20.

## 4. Discussion

**Figure 21:**
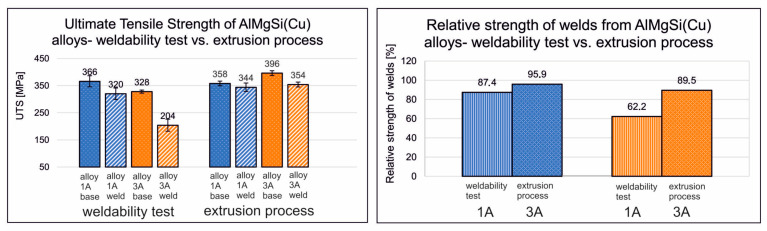
Ultimate tensile strength of AlMgSi(Cu) alloys (on the left) and relative strength of welds from AlMgSi(Cu) alloys (on the right)—according to the weldability test and extrusion process.

**Figure 22:**
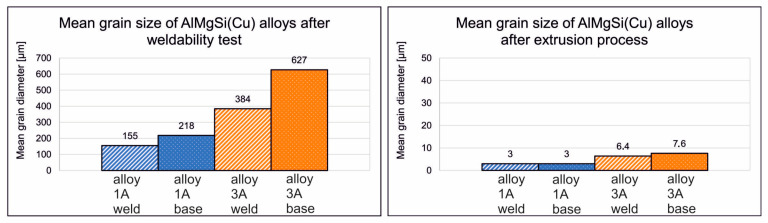
Mean grain size of AlMgSi(Cu) alloys according to the weldability test (**left**) and extrusion process (**right**).

The ultimate tensile strength and the relative strength of AlMgSi(Cu) alloys obtained from the weldability test and extrusion process are presented in Figure 21. Figure 22 shows the mean grain size of AlMgSi(Cu) alloys according to the weldability test and extrusion process. A clear relationship between the grain size and weldability during extrusion can be seen, particularly for weldability tests of AlMgSi(Cu) alloys, where a much larger grain size was obtained for alloy 3A, which resulted in a decrease in the relative strength of the weld to the level of 62.2%. This may be related to the low cooling rate of this alloy, as shown by the welding temperature and grain growth after ageing. However, these problems were no longer observed for the 3A alloy extruded in industrial conditions, where the product was intensively cooled (precipitated) on the press runway immediately after the metal left the die hole, which resulted in a fine-grained structure of the material and high mechanical properties.

The weldability of an extruded metal is defined as its ability (susceptibility) to form a permanent bond between its strands, previously separated at the bridge of the porthole die. Under the conditions of the extrusion of aluminium alloys through porthole dies, the joint occurs in the solid state. The welding of these materials during extrusion on porthole dies is therefore a process of adhesive bonding of solids, occurring in the welding chamber of porthole dies under conditions of hydrostatic pressure and elevated temperature. When the distance between the surface layers of the solids being joined reaches the value of interatomic distances, free electrons can pass from one body to the other, forming micro-joints. The high temperature accompanying the plastic deformation process is the cause of the diffusion of atoms, which leads to the formation of permanent local connections called junction bridges. This phenomenon is also favoured by the high hydrostatic pressure in the joint area. Thus, both of the above-mentioned technological parameters in the extrusion process favour the production of high-quality, high-strength longitudinal welds in extrudates.

**Figure 23:**
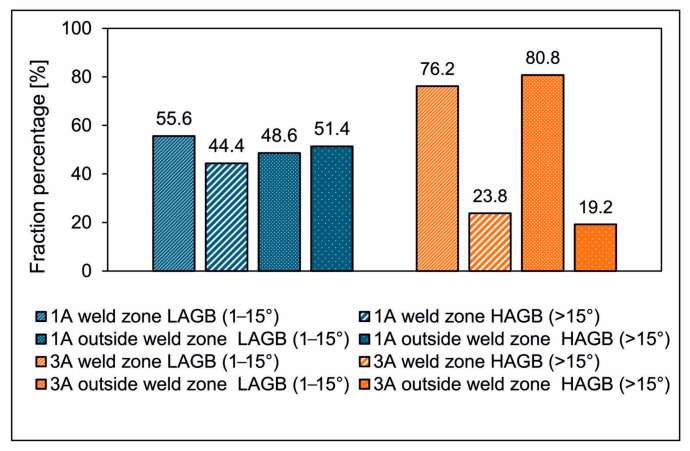
Results angles in and outside the weld zone for profiles extruded from AlMgSi(Cu) alloys 1A of statistical analysis of misorientation and 3A.

Figure 23 compares the percentage of low-angle grain boundaries (LAGB, 1–15°) and high-angle grain boundaries (HAGB, >15°) in two profiles extruded from 1A and 3A alloys. The data are presented both for the weld zone and outside the weld zone. The profile extruded from the 1A alloy has a more balanced distribution between LAGB and HAGB both inside and outside the weld zone. The percentages for LAGB and HAGB are relatively close in both regions. On the other hand, the profile extruded from alloy 3A shows a dominance of LAGB in both regions. In alloy 1A, the proportion of LAGB in the weld zone is approximately 55.6%, whereas the proportion of HAGB is approximately 44.4%. In the area outside the weld zone, the proportion of HAGB is higher and is approximately 51.4%. In the case of the extruded alloy 3A profile, low misorientation angles dominate both in the welding area and outside the welding area. In the weld zone, the proportion of LAGB is about 76.2%, while outside the weld zone the proportion of LAGB is higher and is about 80.8%.

High-angle grain boundaries (HAGBs) play a critical role in influencing the mechanical properties and performance of materials. They act as barriers to dislocation movement, potentially increasing the ductility and toughness of the material. This can improve the weld’s resistance to crack propagation, particularly under stress. In addition, HAGBs facilitate better strain accommodation within the material, reducing the risk of premature failure [31]. In general, more HAGBs in the weld zone are beneficial for improving the mechanical properties of a weld, particularly in terms of toughness and resistance to plastic deformation.

The proposed device for assessing weldability largely takes into account the actual conditions that occur in the real extrusion process through porthole dies. To date, this is the best way to assess the weldability of airless metals and alloys with a quantitative description of the stress temperature conditions. Analysing the conditions of the real extrusion process through porthole dies, it can be concluded that the proposed device does not fully take into account the initial deformation that is realised in the inlet channels of the porthole die during the extrusion process, or the ideal interaction of the frictional forces of the flowing metal through the die. In the real extrusion process, the active surface area of the metal strands being joined is larger and more developed. In fact, these factors favour the weldability of the metal planes, so the weldability result achieved with the proposed device is underestimated compared to the likely results of the real process. In addition, a certain difference in the cooling (precipitation) conditions of the aluminium alloy after welding can be found between the weldability test and the real extrusion process, to the disadvantage of the weldability test. This is due to the more difficult and time-consuming release of the samples from the tool cassette after the weldability test, which has the effect of achieving a lower degree of precipitation of the alloy compared to the precipitation of the alloy immediately on the press run-out. This affects the lower-strength properties of the alloy after weldability tests and final artificial ageing. On the other hand, in the extrusion of aluminium alloys, the hard and brittle oxide surface layers crack and separate, and the surfaces of the pure base metal penetrate into the gaps between the fine oxide fragments, forming metallic bonds. Thus, here, they act to the detriment of weldability, but this only applies to the first stage where the oxide fragments come from the top layer of the ingot and most of them are extruded in a short section of the profile and cut off as process waste. Overall, in the practice of extruding aluminium alloys, the weldability is higher than that obtained from the weldability experiment on the proposed device. Even though the weldability result from the proposed device is at an average level, we are still confident that the mechanical properties of the weld in the extruded section will be sufficiently high.

## 5. Conclusions

Based on weldability testing and industrial verification in the extrusion process of profiles from AlMgSi(Cu) alloys, the following conclusions can be drawn:

1. A new original laboratory method of predicting the seam weld quality of AlMgSi(Cu) extrudates was proposed and positively verified in industrial extrusion trials. This allows the stress temperature conditions of metal welding to be modelled accurately during the extrusion of hollow sections using porthole dies. Crucially, it allows the actual conditions of metal welding to be reproduced without air, as with the welding chamber of porthole dies. This method makes it possible to determine the weldability of a metal during extrusion by determining the so-called relative weld strength, i.e., the strength of a welded sample relative to the strength of a non-welded sample of the base material. The values determined by this method for the minimum welding temperature and minimum welding stress necessary to produce a high-quality metal bond in the welding chambers of porthole dies can effectively support the design of extrusion dies using numerical FEA methods. In practice, this can mean minimisation of the number of costly attempts to implement the extrusion die in technological practice. Research revealed that this method is very rigorous for the weldability of the metal during extrusion, and the results obtained are generally somewhat underestimated, as they does not take into account the factor of pre-plasticisation of the metal in the inlet channels of the porthole die, which is beneficial for the weldability of the metal.
2. Investigations into the material structure and mechanical properties showed that, even with a relatively high Cu content in AlMgSi alloys (above 1.2%), it is possible to obtain high-strength longitudinal welds in extruded profiles. Alloy 1A with a Cu content of 0.61% is characterised by the highest weldability during extrusion, with the relative strength of weld being at the level of 96% as a result of the fine-grained structure, which confirmed the result obtained in the weldability tests: 87% for a welding temperature of 550 °C and a unit welding pressure of 350 MPa. In the case of alloy 3A with a Cu content of 1.22%, high weldability in the extrusion process was also obtained at the level of 89% (in this case, a few larger grains were observed compared to those in alloy 1A), which may indicate the formation of a high-strength weld in the extruded product. For both alloys 1A and 3A, no significant variation in structure or chemical composition was found between the weld zone and the rest of the area, although slightly more high-angle grain boundaries were observed for alloy 3A, which may indicate the slightly lower resistance of this alloy to crack propagation, particularly under stress. In addition, high values of HAGBs for alloy 1A facilitate better strain accommodation within the material, reducing the risk of premature failure, which indicates better weld quality for this alloy.
3. A Cu content in AlMgSi alloys in the range of up to 1.2% is not an obstacle to obtaining a high-strength extruded product. Another aspect of the product quality that should also be taken into account relates to the quality of its surface. This can be effectively enhanced by cooling the die with liquid nitrogen, which also has a positive effect on maximising the metal exit speed, improving the efficiency of the production process of extrusion of AlMgSi alloys with above-standard Cu addition.

## Figures and Tables

**Table 1:** Chemical composition of the samples from AlMgSi(Cu) alloys, in mass per cent.

Alloy Denotation	Si	Fe	Cu	Mg	Cr	Zn	Ti	Zr
AlMgSi(Cu) alloy 1A	1.04	0.05	0.61	0.68	0.25	0.01	0.02	0.15
AlMgSi(Cu) alloy 3A	1.21	0.06	1.22	0.80	0.41	0.01	0.02	0.15

**Table 2:** Results of DSC trails for AlMgSi(Cu) alloys after casting and homogenisation.

Alloy	Solidus Temperature, °C	Incipient Melting Heat, J/g
AlMgSi(Cu) alloy 1A	544.0	0.61
AlMgSi(Cu) alloy 3A	509.2	0.21
AlMgSi(Cu) alloy 1A (homogenised)	596.1	0.13
AlMgSi(Cu) alloy 3A (homogenised)	574.6	1.00

## Data Availability

The original contributions presented in the study are included in the article, further inquiries can be directed to the corresponding author.
